# Medical Practices and Attitudes of Dual-Licensed Medical Doctors in Korea

**DOI:** 10.1155/2013/183643

**Published:** 2013-02-05

**Authors:** Jiseon Ryu, Byunghee Choi, Byungmook Lim, Sina Kim, Youngju Yun

**Affiliations:** ^1^School of Korean Medicine, Pusan National University, Yangsan-si, Gyungnam, Republic of Korea; ^2^Korean Medicine Policy Research Center, Korea Institute of Oriental Medicine, Yuseong-gu, Daejeon 305-811, Republic of Korea

## Abstract

Unique dual medical system in Korea has resulted in the emergence of dual-licensed medical doctors (DLMDs) who have both traditional Korean medicine (KM) and Western medicine (WM) licenses. There have been few studies on DLMDs in spite of their growing number and importance within the medical system. We surveyed the current status and attitudes of DLMD to assess their role in integrative medicine. Questionnaires were administered to the members of the association of DLMD. Data from 103 DLMD were collected and statistically analyzed. 41.4% of DLMD were copracticing both WM and KM at a single clinic, preferring the WM approach for physical examinations, laboratory tests, and education for patients—and the KM approach for treatment and prescription. Musculoskeletal, gastroenterologic, and allergic diseases were considered to be effectively treated with co-practice. DLMD highly agreed on the efficiency of copractice for disease control and patients' satisfaction. On the other hand, they regarded the lack of health insurance coverage for copractice and increased medical expenditure as major problems in providing co-practice. To expand the role of DLMD as mediators of integration in primary health care, the effectiveness of their co-practice should be evaluated and a corresponding health insurance reimbursement system should be established.

## 1. Introduction

Though the National Center for Complementary and Alternative Medicine declared that complementary and alternative medicine (CAM) is not to be considered an integral part of conventional western medicine (henceforth abbreviated “WM”) at present [1], since the introduction of CAM, the terms and concepts of CAM have gradually been integrated into mainstream medicine in many Western countries; likewise disease-centered biomedicine has shifted to holistic patient-centered medicine [2–5]. An increasing number of physicians in Western countries have interest in studying and practicing CAM nowadays [6–9], and many medical schools have included CAM-related content in their compulsory curriculum in the United States [10].

Some Asian countries, however, where traditional medicine exists as a whole medical system, such as Ayurveda or traditional Chinese medicine (TCM), possess their own dedicated and independent medical system due to idiosyncratic historical factors [11]. For example, in China and Taiwan, TCM and WM exist harmoniously and function complementarily while their integration is an ongoing project supported nationally [12, 13]. Ayurveda in India is practiced conventionally and independently from WM on a national scale [11], and recently the government set more rigorous standard requirements for Ayurvedic education and practice than in the past [14, 15].

Korea has a unique dual system where WM and traditional medicine exist on equal terms with exclusive practice boundaries. Traditional Korean medicine (referred to in this paper as “KM”) is not regarded as CAM, but as a part of conventional medicine, mainly due to having its own education and licensing system since the 1950s. Though this kind of system has been advantageous in preserving traditional medicine, it has given rise to institutionalized conflict between the two medical disciplines [16]. In spite of the conflict, KM doctors have tried to combine KM and WM since the inception of KM hospitals in the 1970s; and dual-licensed medical doctors (DLMDs) who had both KM and WM licenses began to emerge and their ranks have grown steeply during the 2000s. Since there have not been special license examinations or integrated education programs such as those in Taiwan and China, it is even more difficult to become DLMD in Korea. The portion of DLMD among medical doctors at large is much smaller than that of Taiwan [12] and the number of an DLMD is around 200 for now. Nevertheless, they draw more and more attention and are expected to assuage the conflicts and to mediate between KM and WM. 

CAM trained medical doctors in Western countries are regarded as mediators in the integration of distinct and different modalities, and studies on their role and attitudes are emphasized [2, 12, 17]. There have been some studies on the attitudes of WM or KM doctors towards the cooperation of KM and WM [18–22]. The studies, however, on DLMD are rare [23]. In this study, we introduced the current situation of DLMD in Korea, investigated their experiences with cooperative practices, and discussed their roles in the future. 

## 2. Methods

### 2.1. Sample and Data Collection

This study is a cross-sectional survey. The questionnaires on the characteristics of the medical practices and attitudes toward copractice were developed and administered to both DLMD and medical students who were already KM or WM doctors and preparing to obtain a second medical license. After obtaining informed consent, the questionnaires were sent by e-mail based on the members' information received from the association of DLMD. The questionnaires were sent twice in January 2011 to 190 members whose e-mail addresses had been verified.

### 2.2. Questionnaire

The questionnaire consists of questions about the following topics: demographics, motive for obtaining dual medical licensure (DML), medical practice, and attitude toward co-practice of the two medical disciplines. Questions about the topic “medical practice and attitude” were measured mainly with 5-point Likert scale (1 = strongly disagree, 3 = neutral, and 5 = strongly agree).

Some terms were defined as follows so as to meet the purpose of this study. “DLMD” refers to those who already have obtained the other license additionally after having obtained a WM or KM license, or students who are preparing for their second license. We included students (prospective DLMD) because they are supposed to obtain DML in the near future, and many of them were doing part time practice and identified themselves as DLMD. “DLMD duration” refers to the duration (in years) from the acquisition of the second license to the moment of participating in the survey. Those who answered as students were assigned a value of 0. “Co-practice” represents the practice in which a single DLMD combines KM and WM procedures or drugs for any given patient. In contrast, “cooperation” denotes the same practice except done by two practitioners—a WM doctor and a KM doctor. 

### 2.3. Statistics

SPSS 18.0 software was used for statistical calculations. Missing values were clearly presented in the results of each question. Response percentage was presented for questions with no responses or questions with multiple responses ranging from 1 to 5 answers.

*χ* ^2^ test and *t*-test were used in order to compare the characteristics and attitudes of participants, and 95% confidence interval or “mean ± SD” was indicated.

## 3. Results 

Among the 190 members of the Association of DLMD receiving the questionnaire, 103 replied within 4 weeks (54.2% response rate). The general characteristic of respondents is presented in Table 1. We made two-group designations to classify the participants: “KM-based DLMD” and “WM-based DLMD.” KM-based DLMD refers to who obtained KM doctor licenses first, while WM-based DLMD are those who first obtained WM doctor licenses.

Most respondents were male (82.5%); 24.5% were students; and the average age was 40 years old (40.35 ± 8.373). Mean age of WM-based DLMD was approximately 5 years higher than that of KM-based DLMD. More than 60% of DLMD replied that they obtained DML within the last five years. As for DLMD duration, the most frequently occurring response among the WM-based group was less than 5 years (48.3%), whereas KM-based DLMD were most likely students preparing for DML (45.2%). 41.4% of respondents have opened clinics where they practiced both WM and KM at a single site (hereafter, such a site will be called a “WM-KM clinic”). Nearly half of the WM-based DLMD (48.3%) and 27.6% of KM-based DLMD were working in WM-KM clinics. The percentage of respondents who have obtained or are scheduled to obtain board certification as WM specialists (37.4%) is greater than that of those having obtained or pursuing the KM one (6.9%). The percentage of KM-based DLMD pursuing or already having obtained WM specialist qualifications is significantly higher than that of their WM-based counterparts. 

The values for motives for obtaining DML were measured with a 5-point Likert scale and the average was calculated (Table 2). All of the means were close to “agree.” “I have been interested in using KM (WM) modalities in my practice.” scored the highest (4.17 ± 0.071), followed by “I thought obtaining DML would give me a competitive advantage over other doctors” (3.78 ± 0.086), and “I wanted to formulate a new medical discipline integrating both WM and KM” (3.70 ± 0.098). WM-based DLMD showed the same order of strength in the motives as the whole group. However, there was a significant difference (*P* < 0.01) in the responses for the question “I thought WM (or KM) by itself has limitations in diagnosis and treatment” between KM-based and WM-based DLMD. KM-based DLMD more strongly agreed (3.79 ± 0.645) than WM-based DLMD (3.25 ± 1.092), and consequently, it has turned out to be the second most important motive in the KM-based DLMD.

**Figure 1:**
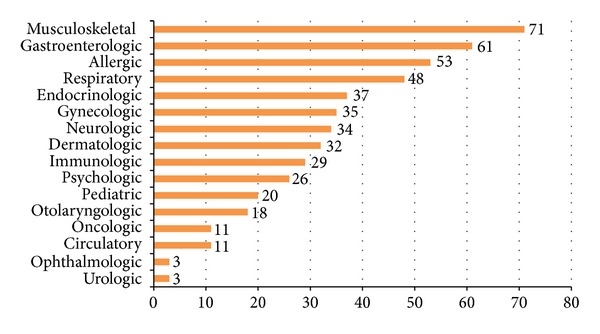
Disease conditions considered to be most effectively treated with copractice by DLMDNote. Multiple response(s): *n* = 103.

The disease condition considered to be the most effectively treated with copractice was musculoskeletal disease (71 responses), followed by gastroenterologic (61 responses) and allergic disease (53 responses) in multiple responses (Figure 1).

**Figure 2:**
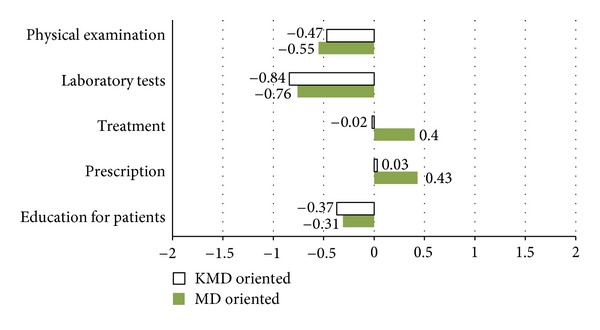
Use of WM and KM modalities in DLMD' practice. Note: 5-point Likert scale (−2 = strong WM approach, 0 = both equally, and 2 = strong KM approach).

We asked about the use of WM and KM modalities in usual practice with 5-point Likert scale (−2 = strongly WM approach; 0 = both equally; 2 = strongly KM approach) (Figure 2). Participants have preferred the WM approach over the KM approach for “physical examinations”, “laboratory tests,” and “education for patients,” while they preferred the KM approach slightly over the WM approach for “treatment” and “prescription,” especially in KM-based DLMD. WM-based DLMD preferred WM modalities for “treatment” (−0.02 ± 1.239), while KM-based DLMD more often used KM modalities (0.40 ± 1.270). The two groups showed opposite, albeit not statistically significant, tendencies.

**Figure 3:**
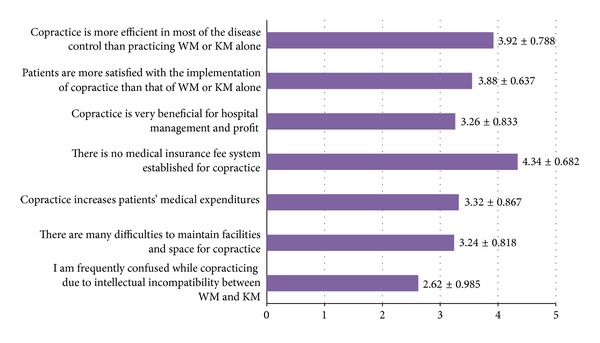
Attitudes of DLMD toward copractice of WM and KM. Note: 5-point Likert scale (1 = strongly disagree, 3 = neutral, and 5 = strongly agree).

Figure 3 shows that DLMD generally took a positive view of the effect of co-practice. They thought that co-practice is more efficient in most of disease control (3.92 ± 0.788) and that patients were more satisfied with co-practice than WM or KM alone (3.88 ± 0.637). On the other hand, they identified some problems in providing co-practice: lack of health insurance coverage for co-practice (4.34 ± 0.682); increased patient's medical expenditures (3.32 ± 0.867); and difficulties in maintaining facilities and space for co-practice (3.24 ± 0.818). Intellectual incompatibility between WM and KM does not seem to be a great difficulty in co-practice (2.62 ± 0.985).

## 4. Discussion

Although KM shares a common origin with TCM, Korea has developed distinctive traditional disciplines and practices [24]. KM is not regarded as CAM in Korea, but rather as a subgroup within conventional medicine, legally speaking. However, many of the KM modalities are not covered by the national health insurance system yet, and KM comprises only a small portion of national insurance expenditures—around 4% of the total. While the medical system of China allows TCM doctors to freely use WM drugs and examinational instruments, in stark contrast, the use of WM modalities by KM doctors is prohibited completely by medical law in Korea. The number of DLMD in Korea has risen since the late 1990s and has rapidly increased during the 2000s [23]. According to the official government statistics in 2010, the total number of WM doctors was 101,307; KM doctors numbered 19,055; and DLMD 206 [25]. Rising interest in the rival discipline can be attributed to the following factors: (1) increased interest in cooperation and the number of cooperating hospitals since the 1990s [26]; (2) the steep climb in the number of doctors, resulting in intense competition; and (3) regulations limiting KM doctors' practices. 

Compared to Taiwan, where 7.6% of WM doctors are DLMD [12], the number of DLMD is still very small in Korea. DLMD constitute approximately 0.2% and 1%, respectively, of WM doctors and KM doctors, at large. Nevertheless, the recent increase in the number of DLMD has become a catalyst for legal and systemic change, resulting in the 2009 legislation making provisions for WM-KM clinics run by DLMD. Before this legislation, DLMD were not allowed to practice WM and KM simultaneously in one clinic, so they had to exclusively choose one or the other for their practice. There are still limitations on DLMD' practicing both modalities in hospital settings. 

### 4.1. Current Status

85.3% of the respondents obtained DMLs in less than 10 years as shown in Table 1, which means many have done it recently or are scheduled to. As stated in the preceding study, the number of DLMD is expected to increase continuously [23]. The percentage of WM-based DLMD was 58.8% and the student portion among them was 10.0%. In comparison, the percentage of KM-based DLMD was 41.2% and students constituted 45.2% of them. The number of KM-based DLMD is growing faster than WM-based DLMD. This implies that KM-based DLMD can become a major constituent of DLMD in the not-too-distant future. The percentage of DLMD that are present or prospective WM specialists is 37.4%, which is significantly lower than the percentage of specialists among WM doctors at large—around 70% in 2010. The number of KM specialists is also lower than that of KM doctors as a whole—around 10% in 2010 [25]. We can conservatively predict steady growth in the number of qualified medical specialists among DLMD. 

Considering that approximately 41% are working or willing to work in WM-KM clinics notwithstanding the fact that the law was legislated only 2 years ago, the number of DLMD working in WM-KM clinics is expected to increase as well. Hospitals and clinics established by DLMD will have a competitive advantage over other medical institutions; and if the ranks of DLMD grow, they will position themselves as a third classification of medical doctors within the dual medical system of Korea. This change may accelerate discussion on the topic of integrated medicine, particularly when governmental efforts toward integration are not enough to produce good results.

### 4.2. Practice of DLMD

The disease conditions considered to be the most effectively treated with co-practice by DLMD were shown to be musculoskeletal, gastroenterologic, and allergic diseases—which are common in primary health care. This result is similar to the previous study [26], where doctors working in cooperative hospitals indicated that musculoskeletal and immunologic diseases are more effectively treated with cooperation than other disease categories. By contrast, this result is different from other studies on the attitudes of doctors toward cooperation, where cerebrovascular (circulatory) disease ranked the highest [18, 21]. We assume that since our respondents were mainly working at local clinics, they were unlikely to indicate “cerebrovascular disease,” the majority of which is observed in inpatients.

As for the results of the use of WM and KM modalities in practice, there exist different tendencies according to the areas of practice. The reason for WM being preferred for examinations and laboratory tests is that KM doctors are legally restricted from using diagnostic devices, thus having limitations in examination and diagnosis. Moreover, it seems that participants regarded using WM terms and approach as advantageous in explaining prognosis and progress during education for patients. On the other hand, they showed preference for KM modalities in the treatment and prescription process. This seems most likely due to the fact that KM has various treatment modalities such as acupuncture, moxibustion, cupping, chu-na, and herbal medicine. Similar results are shown in Lee and Yoo's study about attitudes of WM and KM doctors toward cooperation. KM doctors showed high trust in WM laboratory tests, diagnostic tools such as X-ray, CT, MRI, and so forth. In contrast, WM doctors highly valued acupuncture, moxibustion, cupping, and constitutional discerning tools [21].

### 4.3. Cognition and Attitudes of DLMD

This study also investigated DLMD' attitudes such as motives for obtaining DML and merits and difficulties related to co-practice, which seems important for their prospects.

As for the motives for obtaining DMLs, interest in using other medicinal modalities (4.17 ± 0.719) scored the highest; and limitations of each modality scored relatively low (3.48 ± 0.965) as a whole and among the WM-based DLMD, however, KM-based DLMD more strongly agreed to the limitations of KM. As we mentioned before, these results seem to reflect the legal restrictions on using WM devices by KM doctors.

The greatest difficulty related to co-practice seems to come from discrepancies between the current medical laws and the insurance system. In the Korean national health insurance system, WM and KM each has its own reimbursement system. Since a reimbursement system for co-practice is yet to be established, a DLMD who has used both WM and KM modalities can only be reimbursed for the predominant treatment, which increased out-of-pocket expenses for the patient. 

By contrast, intellectual incompatibility between WM and KM was not considered to be a great difficulty in co-practice (2.62 ± 0.098). This is one of the major findings of our study and a significant divergence from other research pertaining to doctors' attitudes toward cooperation. Other studies identified differing approaches to disease [19–21] and intellectual incompatibility in clinical practice [18] as the greatest difficulties and the reasons for poor cooperation. This means that these aforementioned factors can become obstacles when cooperation is only a parallel implementation of WM and KM modalities, but these hindrances diminish when a single individual practices the two medical modalities. This implies that one of the keys to resolve the conflict between WM and KM lies in training more professionals like DLMD via diverse integrated education courses. 

### 4.4. Limitations and Achievements

One limitation of this study is that we could not survey the whole population of DLMD. Our study included 25 prospective DLMD and 78 present DLMD—around 38% of all present DLMD. Nevertheless, this study is the first to investigate DLMDs' status, attitudes, and perspectives, which may prove invaluable insofar as concrete implications for roles and prospects of DLMD within the Korean medical system may be drawn. Further study soliciting responses from all DLMD would be required to investigate their actual co-practice in detail and formulate an integrative practice model that would be practical and efficient. Comparative effectiveness research on their practice would be also needed for reimbursement by the national health insurance system. 

## 5. Conclusion

Korea faces a less favorable situation than some other countries when it comes to integrating medicine due to its exclusionary, dichotomous medical system. Even though the number of DLMD is still very small, their co-practice can be regarded as a viable way to deal with the situation. Our study suggests that misunderstanding and conflict between different approaches, which are somewhat inevitable between WM and KM doctors, can be mitigated by DLMD. Our study also shows a majority of DLMD have treated common diseases in local clinics by implementing a unique practice model combining modalities of both WM and KM. It is certain that the experiences and suggestions of DLMD will become valuable in the process of integrating medicine especially in primary health care. Development of diverse integrated education courses for both disciplines could accelerate the integration by increasing the number of DLMD-like medical professionals. To expand the role of DLMD as mediators of integration in primary health care, the effectiveness of their co-practice should be evaluated and a corresponding health insurance reimbursement system should be established as soon as possible.

## Figures and Tables

**Table 1:** Characteristics of respondents.

	Total	WM based	KM based	*χ* ^2^ or *T*
	*n* = 103	*n* = 60	*n* = 43	
Gender	(*n* = 103)	(*n* = 60)	(*n* = 43)	
Male	82.5%	85.0%	79.1%	0.611
Female	17.5%	15.0%	20.9%	
Age				
Mean (±SD)	40.35 (±8.373)	42.13 (±6.721)	37.86 (±9.795)	2.627*
DLMD duration (yr)	(*n* = 102)	(*n* = 60)	(*n* = 42)	
0 (student)	24.5%	10.0%	45.2%	20.371***
≤5	40.2%	48.3%	28.6%	
6–10	20.6%	26.7%	11.9%	
11–15	10.8%	13.3%	7.1%	
>5	3.9%	1.7%	7.1%	
Mean (±SD)	4.74 (±5.667)	5.23 (±6.721)	4.02 (±7.141)	1.061*
Work place	(*n* = 87)	(*n* = 58)	(*n* = 29)	
WM- KM clinic	41.4%	48.3%	27.6%	11.201*
WM institution	26.4%	29.3%	20.7%	
KM institution	21.8%	19.0%	27.6%	
Others	10.3%	3.4%	24.1%	
WM specialist (present or prospective)	(*n* = 99)	(*n* = 59)	(*n* = 40)	
Yes	37.4%	27.1%	52.5%	6.561*
KM specialist (present or prospective)	(*n* = 101)	(*n* = 59)	(*n* = 42)	
Yes	6.9%	3.4%	11.9%	2.758

*P < 0.05, ***P < 0.001.

**Table 2:** Motives for obtaining dual medical licensure.

	Mean (±SD)			*T*
	Total	WM based	KM based	
(1) I have been interested in using KM (WM) modalities in my practice	4.17 ± 0.719	4.23 ± 0.698	4.07 ± 0.745	0.265
(2) I thought obtaining DML would give me a competitive advantage over other doctors	3.78 ± 0.860	3.88 ± 0.796	3.64 ± 0.932	0.176
(3) I wanted to formulate new medical discipline integrating both WM and KM	3.70 ± 0.980	3.81 ± 0.972	3.56 ± 0.983	0.210
(4) I wanted to help mediate between WM and KM	3.64 ± 0.944	3.67 ± 0.886	3.60 ± 1.027	0.723
(5) I thought WM (KM) by itself has limitations in diagnosis and treatment	3.48 ± 0.965	3.25 ± 1.092	3.79 ± 0.645	0.003**

^#^Different set of statements was given to each of the two groups.

To WM-based DLMD: (1) “I have been interested in using KM modalities in my practice.”

(5) “I thought WM by itself has limitations in diagnosis and treatment.”

To KM-based DLMD: (1) “I have been interested in using WM modalities in my practice.”

(5) “I thought KM by itself has limitations in diagnosis and treatment.”

**P < 0.01.

Note: 5-point Likert scale (1 = strongly disagree, 3 = neutral, and 5 = strongly agree).
